# Bisdemethoxycurcumin and Its Cyclized Pyrazole Analogue Differentially Disrupt Lipopolysaccharide Signalling in Human Monocyte-Derived Macrophages

**DOI:** 10.1155/2018/2868702

**Published:** 2018-02-08

**Authors:** Serena Tedesco, Morena Zusso, Laura Facci, Annalisa Trenti, Carlotta Boscaro, Federica Belluti, Gian Paolo Fadini, Stephen D. Skaper, Pietro Giusti, Chiara Bolego, Andrea Cignarella

**Affiliations:** ^1^Venetian Institute of Molecular Medicine, Padua, Italy; ^2^Department of Pharmaceutical and Pharmacological Sciences, University of Padua, Padua, Italy; ^3^Department of Pharmacy and Biotechnology, Alma Mater Studiorum-University of Bologna, Bologna, Italy; ^4^Department of Medicine, University of Padua, Padua, Italy

## Abstract

Several studies suggest that curcumin and related compounds possess antioxidant and anti-inflammatory properties including modulation of lipopolysaccharide- (LPS-) mediated signalling in macrophage cell models. We here investigated the effects of curcumin and the two structurally unrelated analogues GG6 and GG9 in primary human blood-derived macrophages as well as the signalling pathways involved. Macrophages differentiated from peripheral blood monocytes for 7 days were activated with LPS or selective Toll-like receptor agonists for 24 h. The effects of test compounds on cytokine production and immunophenotypes evaluated as CD80^+^/CCR2^+^ and CD206^+^/CD163^+^ subsets were examined by ELISA and flow cytometry. Signalling pathways were probed by Western blot. Curcumin (2.5–10 *μ*M) failed to suppress LPS-induced inflammatory responses. While GG6 reduced LPS-induced I*κ*B-*α* degradation and showed a trend towards reduced interleukin-1*β* release, GG9 prevented the increase in proinflammatory CD80^+^ macrophage subset, downregulation of the anti-inflammatory CD206^+^/CD163^+^ subset, increase in p38 phosphorylation, and increase in cell-bound and secreted interleukin-1*β* stimulated by LPS, at least in part through signalling pathways not involving Toll-like receptor 4 and nuclear factor-*κ*B. Thus, the curcumin analogue GG9 attenuated the LPS-induced inflammatory response in human blood-derived macrophages and may therefore represent an attractive chemical template for macrophage pharmacological targeting.

## 1. Introduction

A key feature of macrophages is the ability to “tailor” their responses according to environmental stimuli. Inflammatory agents such as lipopolysaccharide (LPS) and interferon-*γ* (IFN-*γ*) induce an inflammatory phenotype that promotes T_H_1 effector response and antimicrobial and tumouricidal properties. LPS/IFN-*γ*-activated cells are efficient producers of effector molecules (reactive nitrogen and oxygen intermediates) and proinflammatory cytokines, such as interleukin-6 (IL-6), IL-1*β*, and tumour necrosis factor-*α*, while also inducing upregulation of molecules associated with antigen presentation, such as major histocompatibility complex class II and the costimulatory molecules CD40, CD80, and CD86 [1]. Macrophages with this phenotype are called “classically activated” macrophages or M1. In contrast, stimulation of macrophages with cytokines such as IL-4 or IL-13 leads to an “alternative” activation state or M2, characterized by low levels of inflammatory cytokines such as IL-12 and IL-23, and high levels of the anti-inflammatory cytokine IL-10 as well as of scavenger, galactose-type, and mannose receptors (MR or CD206). In general, IL-4/IL-13-activated cells take part in polarized Th2 responses, parasite clearance, dampening of inflammation, promotion of tissue remodelling, angiogenesis, tumour progression, and immunoregulation [2]. Specific macrophage-targeted therapies are now being moved into clinical investigations. In addition, therapeutic approaches not originally designed as macrophage-oriented or specific affect macrophage activation and polarization [3]. Therefore, it is relevant to identify pharmacological agents affecting the functional activation of human macrophages.

The polyphenol curcumin, the principal bioactive component found in the rhizomes of *Curcuma longa* L., an Asian tropical plant belonging to the ginger family (Zingiberaceae), has long been studied for its various therapeutic properties [4, 5]. The presence of the trans-*α*,*β*-unsaturated carbonyl scaffold (referred as hepta-1,4,6-trien-3-one structural motif), being a Michael acceptor system, is thought to be involved in its mechanism of action [6]. This compound can modulate multiple signalling molecules such as transcription factors, enzymes, and secondary messengers, thereby controlling expression of a variety of genes, and is potentially effective in disease conditions associated with impaired regulation of such signalling pathways [7]. In particular, curcumin reduces LPS-induced activation and release of inflammatory cytokines by microglia and macrophages [8–13]. We and others demonstrated that curcumin and related compounds prevent activation of nuclear factor-*κ*B (NF-*κ*B), a transcriptional factor that regulates many genes involved in the initiation of inflammatory responses [13, 14]. This polyphenol also affects the immunophenotype of LPS-activated human THP-1 macrophages, especially the expression of M1 markers that are modulated through the NF-*κ*B pathway [10, 15]. Interestingly, curcumin not only directly induces polarization of murine RAW264.7 macrophages to the M2 phenotype but also promotes a M1-to-M2 phenotype switch [16]. More recently, Gao et al. [17] demonstrated that curcumin induces macrophage M2 polarization by increasing the production of IL-4 and/or IL-13 in a signal transducers and activators of transcription 6-dependent manner. These findings have been also replicated *in vivo* in experimental models of disease [17, 18]. Based on these observations, curcumin and its analogues represent promising lead compounds targeting immune cell activation.

Toll-like receptors (TLRs) are a family of transmembrane proteins that play a key role in the innate immune system. TLRs generally exist as homodimers (although TLR2–TLR6 and TLR1-TLR2 heterodimers have been reported) and are found on immune cells including macrophages. Ten human TLRs have been identified to date, all of which are expressed in human macrophages at the cell surface or in endosomes [19]. LPS signalling is mediated mainly by TLR4-MD-2 activation and dimerization [20]. Previous studies demonstrated its inhibition by curcumin and its structurally related bisdemethoxycurcumin (GG6), both carrying the hepta-1,4,6-trien-3-one scaffold, whereas the GG6-based pyrazole analogue (GG9) was inactive [13].

This extensive body of literature dealing with the pharmacological effects of curcumin and analogues on immune cell function has, to the best of our knowledge, not been extended to human blood-derived macrophages. This may be relevant due to differences between rodent and human immune responses as well as among the many macrophage cell models used [21, 22]. In addition, poor bioavailability, rapid metabolism, and adverse effects of curcumin observed in some studies [23, 24] have provided the impetus for developing curcumin-based analogues with improved pharmacodynamic and pharmacokinetic properties [25]. The present study was designed to investigate the effects of curcumin and the curcumin-based analogues GG6 and GG9 [13] on IL-1*β* release by activated human monocyte-derived macrophages as well as on the signalling pathways involved. The effects of GG9 on macrophage immunophenotypic marker expression and cell-bound IL-1*β* were also examined by flow cytometry.

## 2. Materials and Methods

### 2.1. Materials

RPMI 1640 was purchased from Lonza (Basel, Switzerland), and antibiotic solution (100 U/ml penicillin and 100 *μ*g/ml streptomycin) was from Invitrogen Inc. (Carlsbad, CA, US). Monoclonal anti-human I*κ*B-*α*, phospho-p38 mitogen-activated protein kinase (MAPK), and glyceraldehyde-3-phosphate dehydrogenase (GAPDH) antibodies were from Cell Signalling (Danvers, MA, US). Horseradish peroxidase-conjugated secondary antibodies were from Vector (Peterborough, UK). Anti-CCR2 mAb, anti-CD163 mAb, anti-IL-1*β* mAb, brefeldin, Fix, and Perm buffer solutions were from eBioscience/Affymetrix (Santa Clara, CA, US); anti-CD80 mAb and anti-CD206 mAb were from BD Biosciences Pharmigen (San Diego, CA, US). The cOmplete™ inhibitor cocktail was from Roche Diagnostics (Mannheim, Germany). Fetal bovine serum (FBS), Ficoll-Paque (density 1.077 ± 0.001), Percoll, dexamethasone, curcumin, CLI-095, skim milk powder as well as other analytic grade chemical agents were from Sigma-Aldrich. Ultrapure LPS (LPS-EB), zymosan (ZYMO), Pam3CSK4, and Poly(I:C) were from InvivoGen (San Diego, CA, USA). IL-4, IL-13, and colony stimulating factor-1 (CSF-1) were from ImmunoTools (Friesoythe, Germany). The curcumin analogues GG6 and GG9 were synthesized as described elsewhere [13].

### 2.2. Cell Culture

Human peripheral mononuclear cells (PBMCs) were isolated from deidentified buffy coats provided by the Transfusion Unit at Padua University Hospital. After Ficoll separation, PBMCs were separated using a Percoll density gradient to obtain pure monocytes. The separation of monocytes from lymphocytes was performed with a high-density hyperosmotic Percoll density gradient: the PBMC suspension, containing 150–200 × 10^6^ cells, was layered on top in a 1 : 3.33 ml ratio. After centrifugation at 600*g* for 15 min, cells at the interface were collected and washed with serum-free RPMI 1640, counted, and seeded in 24- or 48-well plates or 60 mm dishes as indicated at a density of 5 × 10^4^ cells/cm^2^ in RPMI 1640 + 10% FBS in the presence of 20 nM CSF-1 [26]. Cells were cultured over 7 days at 37°C and 5% CO_2_, and the medium was changed every 3 days, added freshly.

### 2.3. Protocols of Polarized Activation

At the end of differentiation, macrophages seeded in 60 mm dishes were washed and polarized towards either the M1 phenotype by incubation with LPS (100 ng/ml) or the M2 phenotype by incubation with IL-4 (20 ng/ml) + IL-13 (5 ng/ml), respectively, for 24 h in RPMI + 10% FBS as described [27]. It should be pointed out that commercial sources of LPS are frequently contaminated by other bacterial components, such as lipoproteins, capable of activating also TLR2. In contrast, Ultrapure LPS-EB used here (InvivoGen) only activates TLR4. Test compounds at concentrations used previously (7.5 *μ*M curcumin, 2.5 and 7.5 *μ*M GG9, 7.5 *μ*M GG6, or 1 *μ*M CLI-095) [13] were added 30 min before polarized activation. In selected experiments, unstimulated (M0) macrophages were challenged for 24 h with either 100 nM dexamethasone or 7.5 *μ*M GG9.

### 2.4. Immunophenotyping

At the end of activation, macrophages were harvested by gently scraping culture plates with phosphate-buffered saline (PBS) containing 5 mM EDTA, collected in round-bottom tubes for immunolabeling (BD Biosciences) and resuspended at 10^6^/ml in 100 *μ*l PBS with 2% FBS to block Fc receptors. Purity in these cultures was first assessed by staining cells with PE-anti-CD14 (20 *μ*l/10^6^ cells). Macrophages were stained for 30 min in the dark with fluorochrome-tagged monoclonal antibodies against surface CD80-FITC (20 *μ*l/10^6^ cells) and CCR2-APC (10 *μ*l/10^5^ cells) to identify the M1 phenotype and against CD206-FITC (20 *μ*l/10^6^ cells) and CD163-PE (20 *μ*l/10^6^ cells) to characterize the M2 phenotype [27–29]. After incubation, samples were washed and suspended in 250 *μ*l PBS/EDTA, and 10,000 events/sample for each tube were recorded using a FacsCanto II flow cytometer (BD Biosciences). Data were analyzed using FacsDiva software (BD Biosciences). Isotype-matched controls were used as a reference.

### 2.5. Intracellular Cytokine Production

Intracellular cytokine production was also evaluated using flow cytometry. After CSF-1 differentiation, macrophages were stimulated with LPS (100 ng/ml) or other TLR ligands such as zymosan (10 *μ*g/ml), Pam3CSK4 (0.3 *μ*g/ml), and Poly(I:C) (50 *μ*g/ml) for 24 h in RPMI + 10% FBS. Brefeldin (10 *μ*M) was added during the last 4 h to disrupt cytokine secretion. At the end of stimulation, cells were harvested by gently scraping the culture plates with 1 ml PBS containing 5 mM EDTA and 2% FBS, fixed with 100 *μ*l 4% paraformaldehyde solution and permeabilized by adding 2 ml Perm Buffer solution containing 0.1% saponin and 0.009% sodium azide. Cells were centrifuged twice at 300*g* for 5 min and incubated in 100 *μ*l Perm Buffer solution in the presence of FITC-anti-IL-1*β* (5 *μ*l/10^6^ cells) for 20 min in the dark. Subsequently, cells were washed and resuspended in 250 *μ*l PBS/EDTA, and 10,000–50,000 events/sample for each tube were recorded.

### 2.6. Western Blot

After polarized activation in 60 mm dishes, cells were washed once with PBS and harvested in 150 *μ*l lysis buffer (50 mM Tris HCl pH 7.4, 150 mM NaCl, 1% NP-40, 25 mM NaF, 0.5% Na deoxycholate, 10% SDS, 1 mM EGTA, 1 mM sodium orthovanadate, 10 mM sodium pyrophosphate, 1 mM phenylmethylsulfonylfluoride and cOmplete protease inhibitor cocktail (Roche). Each sample comprised two pooled plates and was stored at −20°C until further analysis. Protein lysates were heated at 100°C for 5 min to denature proteins, and 40 *μ*g of each sample (protein content being measured using the Lowry method) was loaded onto a 10% SDS/polyacrylamide gel and run at 120 V for 1 h. Proteins were then transferred to a polyvinylidene difluoride membrane, nonspecific binding sites blocked with 5% milk in 1 M Tris HCl pH 7.4, 5 M NaCl, and 0.1% Tween 20 for 1 h at room temperature, washed, and then incubated with specific primary antibodies against I*κ*B-*α*, phosphorylated p38 MAPK, and GAPDH (all 1 : 1000). Membranes were incubated with appropriate horseradish peroxidase-conjugated secondary antibodies (1 : 5000), and immunoreactive bands were detected using enhanced chemiluminescence. Band intensities were normalized to GAPDH and measured using ImageJ software (US National Institutes of Health).

### 2.7. Immunofluorescence

Macrophages were grown on coverslips in 12-well plates and pretreated for 1 h with 7.5 *μ*M curcumin, GG6, GG9, or 1 *μ*M CLI-095 and then stimulated with 100 ng/ml Ultrapure LPS-EB for an additional 90 min. Cells were fixed with 4% paraformaldehyde (pH 7.4, for 15 min at room temperature) and subsequently blocked with 5% normal goat serum/0.1% Triton X-100 in PBS for 1 h as described [30]. Cells were incubated with a mouse anti-p65 antibody (NF-*κ*B p65, 1 : 500; Santa Cruz Biotechnology, Santa Cruz, CA, USA) followed by an Alexa Fluor 555-conjugated anti-mouse secondary antibody (1 : 1000; Invitrogen). Nuclei were stained with 4,6-diamidino-2-phenylindole (DAPI, Sigma), and coverslips were mounted on microscope slides with Fluoromount-G mounting medium (Thermo Fisher Scientific, Milan, Italy). Fluorescent images for p65 staining were captured with a confocal laser-scanning microscope (Zeiss LSM 800; Carl Zeiss AG, Oberkochen, Germany) and analyzed with ZEN 2.0 imaging software (Carl Zeiss AG).

### 2.8. Cytokine Release

Monocytes were seeded in 24-well plates at a density of 10^6^ cells/well and allowed to differentiate for 7 days as described above. Cells were primed using 1 *μ*g/ml LPS in serum-free RPMI for 24 h in the presence or absence of curcumin analogues at concentrations selected based on preliminary experiments. Cell supernatants were collected and stored at −20°C until analysis. Cell lysates were prepared by adding 40 *μ*l lysis solution to each well. The amount of IL-1*β* in the culture medium and cell lysates was assessed using a commercially available ELISA kit according to the manufacturer's instructions (Antigenix America, Huntington Station, NY, USA). The IL-1*β* ELISA assay kit does not distinguish between the inactive 31-kDa precursor (pro-IL-1*β*) and the bioactive 17 kDa mature form (as is the case for all commercially available kits). Standard curves were used to obtain sample IL-1*β* concentrations (pg/ml).

### 2.9. Statistical Analysis

Statistical analysis was performed using Prism software (GraphPad Software Inc., La Jolla, CA, US). Data were expressed as mean ± SEM. *t*-test or ANOVA followed by Bonferroni post hoc test was used for comparison between samples. A *p* value < 0.05 was considered to be statistically significant.

## 3. Results

### 3.1. Effects of the Pyrazole Curcumin Analogue (GG9) on Macrophage Activation Phenotypes

**Figure 1:**
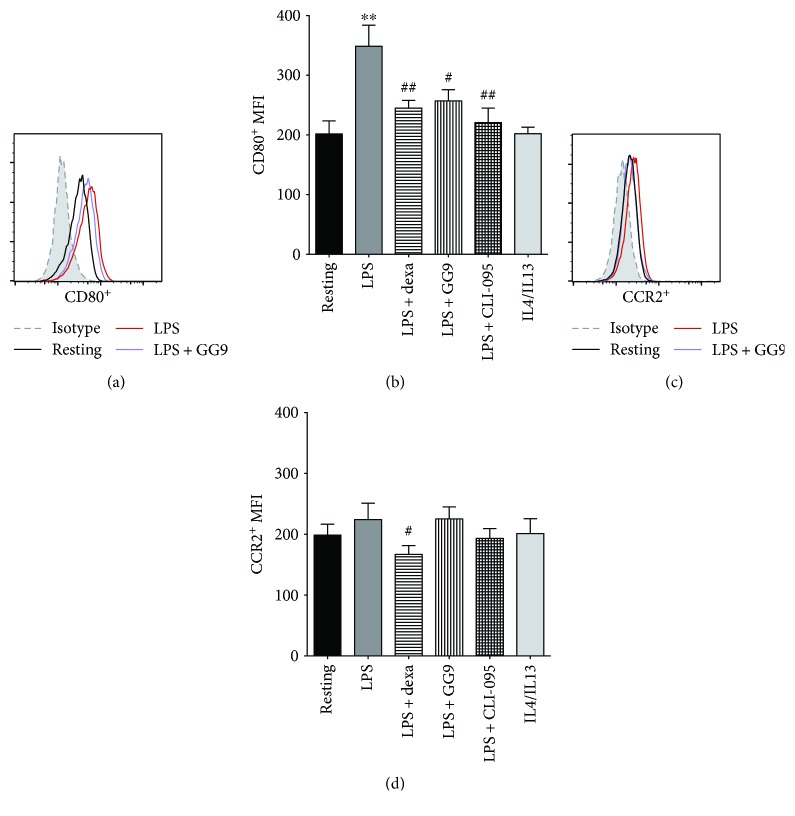
Effects of GG9, dexamethasone (dexa), and CLI-095 on LPS-induced activation of M1 macrophage markers CD80 and CCR2. Cells were pretreated with dexa, GG9, and CLI-095 for 30 min and then polarized using either 0.1 *μ*M LPS (M1) or 20 ng/ml IL-4 plus 5 ng/ml IL-13 (M2) for 24 h in RPMI + 10% FBS as described in Materials and Methods. (a, c) Representative flow cytometry assays for CD80^+^ and CCR2^+^ cells. Bar graphs represent the mean (±SEM) of CD80^+^ (b) and CCR2^+^ (d) mean fluorescence intensity (MFI) from 6 independent experiments. ^∗∗^ *p* < 0.01 versus resting; ^#^ *p* < 0.05 and ^##^ *p* < 0.01 versus LPS.

**Figure 2:**
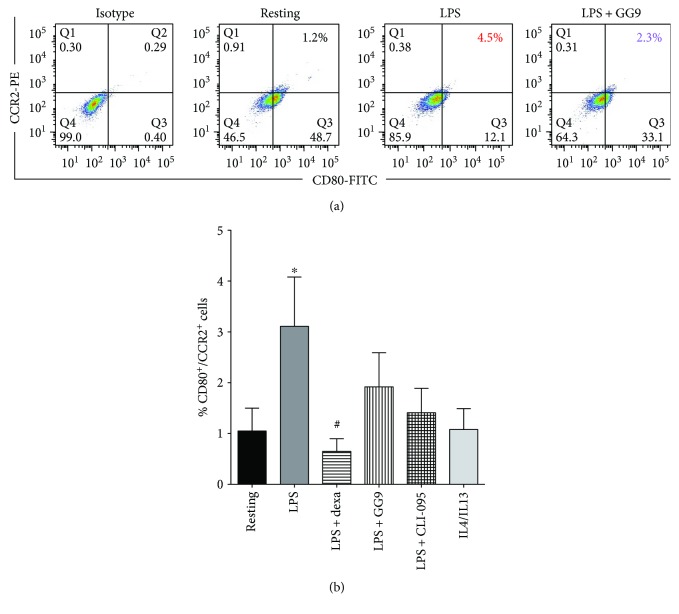
Effects of GG9, dexamethasone (dexa), and CLI-095 on the M1 macrophage phenotype as defined by CD80^+^/CCR2^+^ cells. Cells were activated and treated as described in the legend of Figure 1. Fluorescence quantification dot plots (a) are from a representative experiment. Bars represent the mean (±SEM) of CD80^+^/CCR2^+^cells (b) from 6 independent experiments. ^∗^ *p* < 0.05 versus resting; ^#^ *p* < 0.05 versus LPS.

We then performed flow cytometry experiments to investigate macrophage activation. The yellow color of curcumin and GG6 solutions interferes with the flow cytometer laser detection, and so the two compounds were excluded from this set of analyses. Dexamethasone (dexa) was used as a reference anti-inflammatory agent based on previous work [27]. The possible effects of GG9 at a noncytotoxic concentration on immunophenotypes were compared to those of CLI-095, which blocks signalling mediated by the intracellular but not extracellular domain of TLR4 [31]. Cells were pretreated with dexa (100 nM), CLI-095 (1 *μ*M), or GG9 (7.5 *μ*M) for 30 min, and subsequently incubated for 24 h with fresh medium to obtain resting macrophages (M0) or with either LPS or IL-4/IL-13 to obtain macrophages activated to the M1 and M2 phenotypes, respectively. Representative cell surface CD80 levels are shown in Figure 1(a). Treatment with LPS but not with IL-4/IL-13 for 24 h markedly induced the fraction of cells expressing the M1 marker CD80 (Figure 1(b)) that was significantly suppressed by GG9, dexa, and CLI-095. While the change in CCR2^+^ cells induced by LPS was not significant (Figures 1(c) and 1(d)), dexa but not GG9 or CLI-095 significantly reduced this cell subpopulation compared with resting cells. Pretreatment with dexa significantly prevented the upregulation of M1 (CD80^+^/CCR2^+^) cells induced by LPS (0.7 ± 0.3% versus 3.0 ± 1.0%, *p* < 0.05, *n* = 6; Figures 2(a) and 2(b)), whereas a nonsignificant trend of inhibition was observed for GG9 and CLI-095.

**Figure 3:**
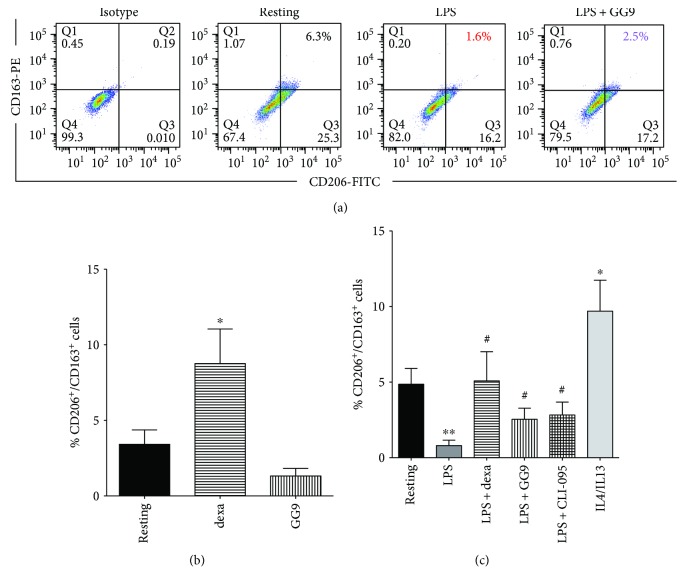
Effects of GG9, dexamethasone (dexa), and CLI-095 on the M2 macrophage phenotype as defined by CD206^+^/CD163^+^ cells. Cells were activated and treated as described in the legend to Figure 1. Fluorescence quantification dot plots (a) are from a representative experiment. (b) Bars represent the mean (±SEM) fraction of CD206^+^/CD163^+^ cells after pretreatment with test agents as indicated from 5-6 independent experiments. (c) Fraction of CD206^+^/CD163^+^ resting macrophages following treatment with test agents for 24 h as indicated. ^∗^ *p* < 0.05; ^∗∗^ *p* < 0.005 versus resting; and ^#^ *p* < 0.05 versus LPS.

We next investigated modulation of the M2 markers CD163 and CD206 (Figure 3(a)) following activation by LPS or IL-4/IL-13. As expected, the fraction of CD206^+^/CD163^+^cells was enhanced in the presence of IL-4/IL-13 compared with resting cells (Figure 3(b)). In contrast, proinflammatory activation with LPS sharply decreased the fraction of M2 macrophages that was restored by dexa treatment (5.9 ± 2.0 versus 1.1 ± 0.4%, *n* = 5). Similarly, GG9 significantly prevented LPS-induced downregulation of CD206^+^/CD163^+^cells (3.4 ± 1.0 versus 1.1 ± 0.4%, *n* = 5; Figure 3(b)), as did CLI-095 (3.4 ± 0.8 versus 1.1 ± 0.4%, *p* < 0.05, *n* = 5). The same pattern was observed in cells expressing each single M2 marker (data not shown). In order to assess a possible direct effect of GG9 on M2 polarization, the experiment was repeated in unstimulated macrophages. Treatment with dexa for 24 h significantly increased the percentage of CD206^+^/CD163^+^ subpopulations with respect to untreated macrophages (8.8 ± 2.2 versus 3.4 ± 0.9%, *p* < 0.05, *n* = 6), as previously published by our group [27]. In contrast, GG9 treatment did not affect the percentage of CD206^+^/CD163^+^ cells (Figure 3(c)).

### 3.2. Effects of Curcumin, GG6, and GG9 on LPS-Induced Signalling

**Figure 4:**
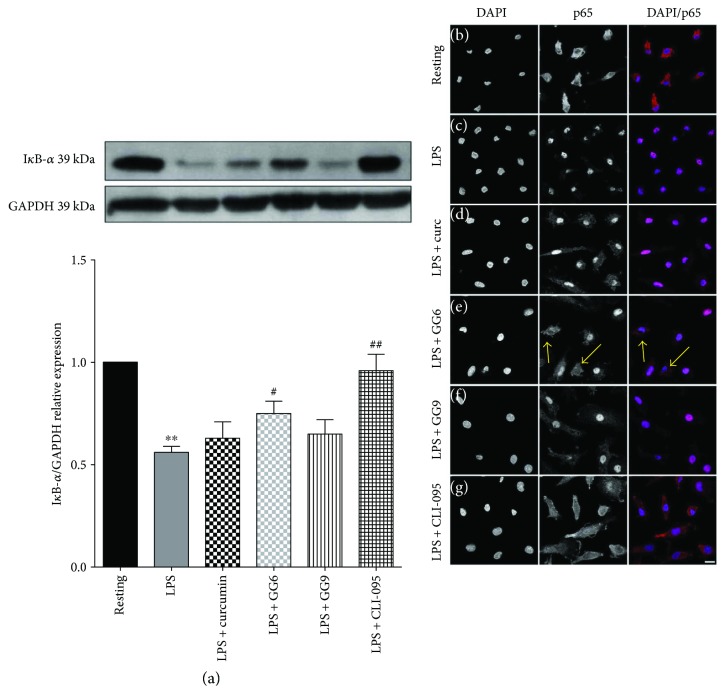
(a) I*κ*B-*α* immunodetection in CSF-1-driven macrophages. Total protein extracts from resting, LPS-stimulated M1, and test agent-pretreated M1 macrophages were assayed after 30 min LPS incubation. GAPDH served as loading control. Bars represent the mean (±SEM) of 4 independent experiments. Results from representative immunoblots are shown. ^∗∗^ *p* < 0.0005 versus resting; ^#^ *p* < 0.05; and ^##^ *p* < 0.005 versus LPS. (b–g) Cells were pretreated with curcumin, GG6, GG9, and CLI-095 before stimulation with LPS, and then processed for NF-*κ*B p65 immunostaining. Representative confocal images showing subcellular localization of p65 in unstimulated and LPS-stimulated macrophages are shown in (b) and (c), respectively. Treatment with curcumin, GG6, GG9, and CLI-095 are shown in (d–g), respectively. Arrows indicate representative cells with attenuated p65 staining. Scale bar: 10 *μ*m; magnification: 63x.

We previously reported that curcumin and GG6, unlike GG9, inhibit LPS-induced activation of NF-*κ*B in primary rat microglia [13]. However, as shown above, GG9 was effective in preventing the increase in the costimulatory molecule CD80, a strong membrane marker of M1 polarization, induced by LPS via transcriptional mechanisms [15, 32]. To identify potential signalling pathways underlying the immunophenotype findings for GG9, we measured I*κ*B-*α* degradation that is required for NF-*κ*B transcriptional activation. In contrast to rat microglia, I*κ*B-α degradation induced by a 30 min challenge with LPS was unchanged by curcumin (Figure 4). Similarly, pretreatment with GG9 did not affect this pathway, whereas GG6 and even more so the inhibitor of TLR4 signal transduction CLI-095 significantly increased I*κ*B-*α* levels compared with LPS alone (Figure 4(a)). We also assessed LPS-induced translocation of NF-*κ*B by p65 immunofluorescence and confirmed that GG6 and CLI-095 were the only compounds affecting the NF-*κ*B pathway in the cell model used in this study (Figures 4(b–g)).

**Figure 5:**
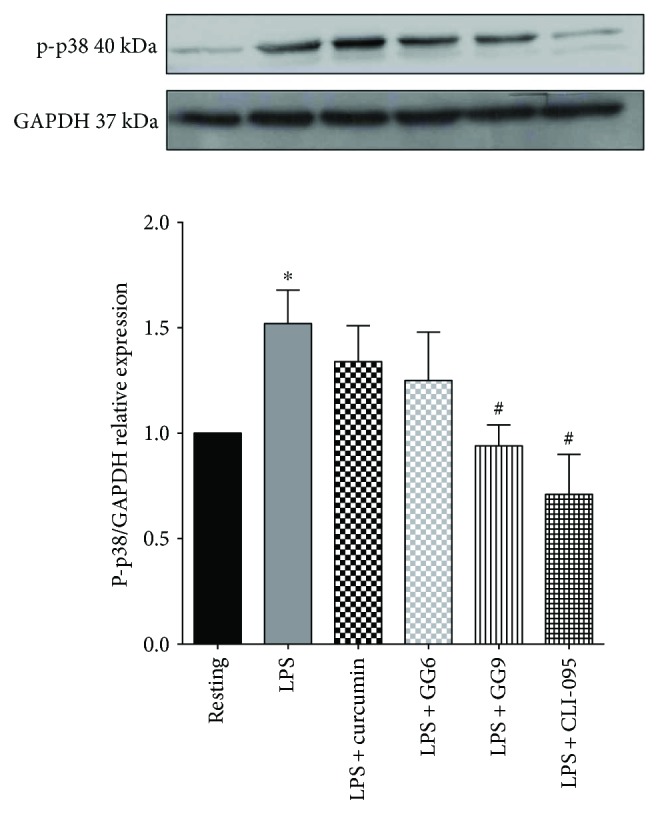
Phospho-p38-MAPK immunodetection in CSF-1-driven macrophages. Total protein extracts from resting, untreated M1, and test agent-pretreated M1 macrophages were assayed after 30 min LPS incubation. GAPDH served as loading control. Bars represent the mean (±SEM) of 6 independent experiments. Results from representative immunoblots are shown. ^∗^ *p* < 0.05 versus resting; ^#^ *p* < 0.05 versus LPS.

Because the p38 kinases are responsive to stresses such as cytokine stimulation and curcumin inhibits p38 MAPK phosphorylation in RAW 264.7 murine macrophages [33], immunodetection of phospho-p38 was performed under similar conditions. While pretreatment with curcumin and GG6, sharing the arylhepta-1,4,6-trien-3-one structural motif, failed to affect LPS-induced p38 phosphorylation, pretreatment with GG9 and CLI-095 significantly reduced p-p38 levels (Figure 5). These data demonstrate the importance of the pyrazole function for the regulation of LPS signalling in human monocyte-derived macrophages.

### 3.3. Effects of Test Compounds on IL-1*β* Production Induced by TLR Ligands

**Figure 6:**
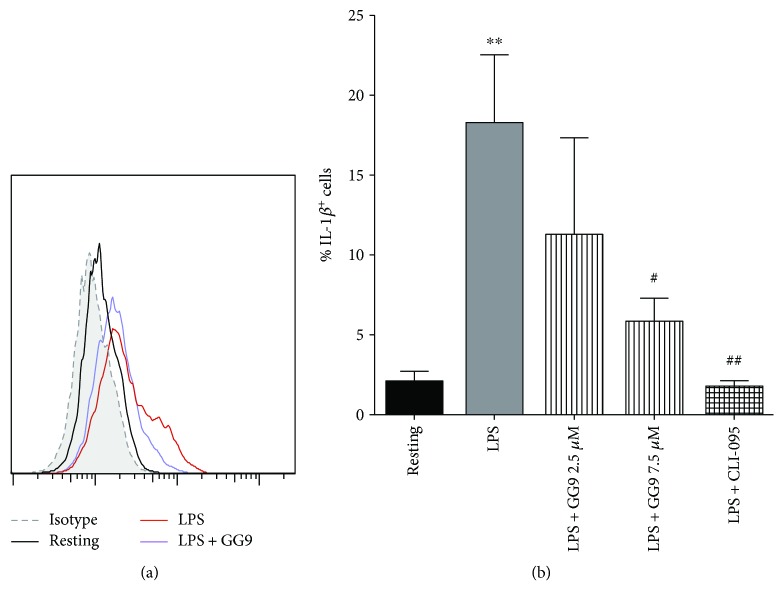
Effects of GG9 and CLI-095 on cell-bound IL-1*β* expression in LPS-stimulated macrophages. After differentiation, macrophages were pretreated with either GG9 or CLI-095 for 30 min, and then incubated with LPS for 24 h at 37°C in RPMI + 10% FBS in the presence of brefeldin (10 *μ*M) over the last 4 h to disrupt cytokine secretion pathways. Prior to flow cytometry analysis, fixed/permeabilized cells were stained with anti-IL-1*β* antibodies. Fluorescence plots are from representative experiments (a), and bar graphs represent the mean (±SEM) of 5 independent experiments (b). ^∗∗^ *p* < 0.01 versus resting; ^#^ *p* < 0.005; and ^##^ *p* < 0.001 versus LPS.

A functional readout of the anti-LPS effect on M1 phenotypic markers as shown by GG9 (Figure 1) is cytokine production. Stimulation of macrophages with LPS results in increased production of proinflammatory cytokines. This was indeed the case for cell-bound IL-1*β* (Figures 6(a) and 6(b)) in LPS-stimulated cells compared with levels detectable in unstimulated cells as measured by flow cytometry. LPS-induced cellular accumulation of IL-1*β* was significantly reduced by pretreatment with both 7.5 *μ*M GG9 and 1 *μ*M CLI-095 (*n* = 5; Figure 6(b)).

**Figure 7:**
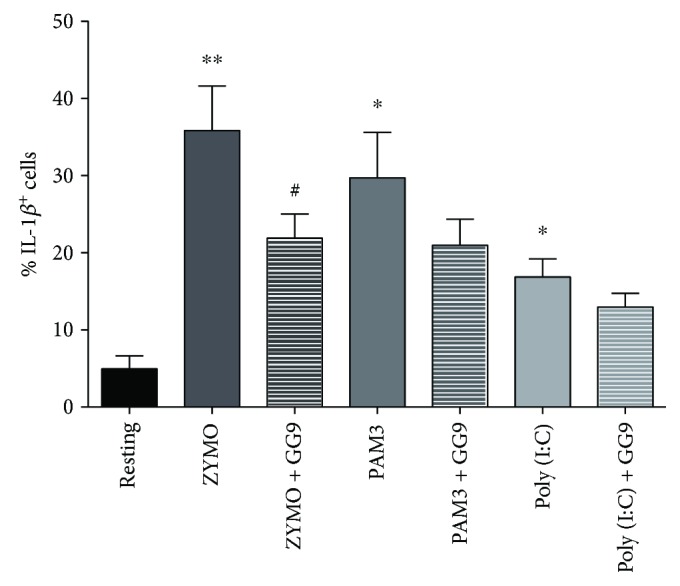
Effect of GG9 on macrophage cell-bound IL-1*β* expression induced by TLR ligands. After differentiation, macrophages were pretreated with GG9 for 30 min, and then incubated with zymosan (10 *μ*g/ml), Pam3CSK4 (PAM3, 0.3 *μ*g/ml), and Poly(I:C) (50 *μ*g/ml) for 24 h at 37°C in RPMI + 10% FBS in the presence of brefeldin (10 *μ*M) over the last 4 h to disrupt cytokine secretion pathways. Prior to flow cytometry analysis, fixed/permeabilized cells were stained with anti-IL-1*β* antibody. Bar graphs represent the mean (±SEM) of 4 independent experiments. ^∗^ *p* < 0.05; ^∗∗^ *p* < 0.001 versus resting; and ^#^ *p* < 0.05 versus TLR agonist.

Because GG9 displayed pharmacological activity on IL-1*β* production in human macrophages as opposed to rat microglia [13], we explored its potential to interfere with additional TLRs that may involve different signalling pathways. The flow cytometry cell-bound IL-1*β* assay was repeated using TLR ligands other than LPS. Zymosan, a yeast TLR2 and TLR2/TLR6 agonist, markedly increased the IL-1*β* ^+^ subpopulation that was significantly reduced by about 40% after pretreatment with GG9 (Figure 7). The TLR3 activator Poly(I:C), a synthetic analogue of double-stranded RNA, was less effective than the TLR2/TLR1 agonist Pam3CSK4 in increasing the IL-1*β* ^+^ subpopulation, which was unchanged after pretreatment with GG9 in either case (Figure 7). Conceivably, GG9 could interfere with TLR2 in the human macrophage inflammatory response.

**Figure 8:**
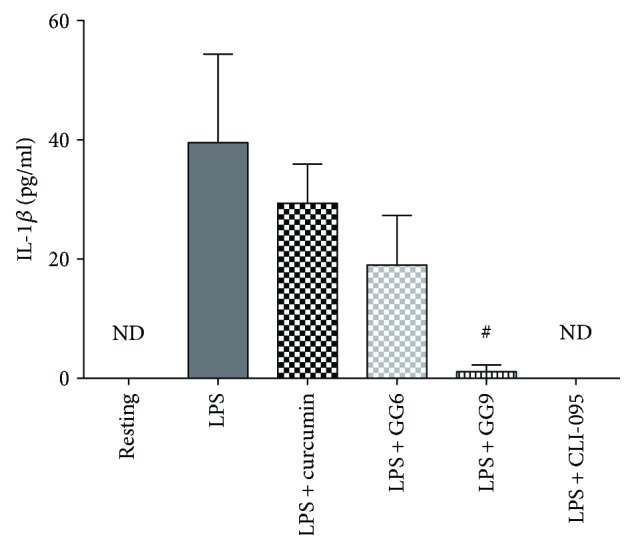
Effects of curcumin, GG6, GG9, and CLI-095 on IL-1*β* release from LPS-stimulated human macrophages. After differentiation, macrophages were pretreated with curcumin, GG6, GG9, and CLI-095 as indicated for 30 min followed by stimulation with 1 *μ*g/ml LPS for 24 h in FBS-free RPMI. Supernatants were collected and analyzed for IL-1*β* release by ELISA. Bar graph represents the mean (±SEM) of 3 independent experiments. ^#^ *p* < 0.05 versus LPS.

Because the functional effects of curcumin and GG6 could not be tested using flow cytometry, we measured IL-1*β* release by LPS-stimulated macrophages (see also [13]). Basal IL-1*β* release by CSF-1-differentiated macrophages in the absence of inflammasome activator was barely detectable by ELISA (data not shown), consistent with recent studies [34]. Stimulation with LPS for 24 h significantly increased the release of IL-1*β* into the medium (39.5 ± 14.8 pg/ml, *n* = 3; Figure 8). Of note, human macrophages release about 10-fold less IL-1*β* than primary rat microglia [9], with noticeable variation among donors. Pretreatment with GG9 and CLI-095 remarkably reduced IL-1*β* release to less than 10% of the LPS level (Figure 8), in line with data in Figure 6. In contrast, the trend of GG6 towards attenuation of LPS-induced IL-1*β* release did not achieve statistical significance, and curcumin induced no apparent effect.

We analysed in more detail the IL-1*β* pathway in polarized macrophages by measuring mRNA levels of the NLRP3 inflammasome. The significant reduction in LPS-induced cellular accumulation (Figure 6) and release (Figure 8) of IL-1*β* following pretreatment with GG9 and CLI was associated with a trend towards increased NLRP3 mRNA levels (Figure S3). To support the potential involvement of the NLRP3 inflammasome in GG9 action, we showed that this agent had no effect on TNF-*α* cellular accumulation, which is independent from inflammasome activation (Figure S4). These data suggest that GG6 and GG9 interfere with differential and possibly redundant inflammatory signalling pathways in human macrophages.

## 4. Discussion

In this study, we investigated whether the effects of the naturally occurring curcuminoids curcumin and bisdemethoxy curcumin (GG6), together with a pyrazole analogue obtained by GG6 cyclization, on macrophage immunophenotype and function as reported in a variety of cell types could be reproduced in human monocyte-derived macrophages. Unexpectedly, curcumin was ineffective against all study endpoints, whereas GG6 and GG9 displayed differential pharmacological profiles that were only partially consistent with those observed in primary rat microglia [13].

Although we were unable to test curcumin and GG6 by flow cytometry due to the yellow color of their solutions, GG9 prevented upregulation of CD80 expression induced by LPS activation [15, 32], thereby attenuating M1 polarization. The effect of GG9 on the CD80^+^/CCR2^+^ subset was, however, not significant (Figure 2), as the fraction of CCR2^+^ cells was unchanged following LPS activation (Figure 1(b)). In contrast with previous studies with curcumin [17], GG9 had no effect on the M2 phenotype in unstimulated cells, but counteracted the decrease in the anti-inflammatory CD206^+^/CD163^+^ subset induced by LPS [27, 29]. These findings suggest that GG9 was able to interfere with transcriptional mechanisms underlying upregulation of costimulatory molecules such as CD80, but not with desensitization and internalization pathways that largely govern surface levels of G-protein-coupled receptors such as CCR2 [35]. In contrast, treatment with dexamethasone reduced the CD80^+^/CCR2^+^ subset and enhanced the CD206^+^/CD163^+^ subset in both LPS-treated and untreated macrophages (Figures 3(b) and 3(c)). This is consistent with findings from several cell models [27, 36, 37], suggesting that dexamethasone, unlike GG9, induces a direct effect on M2 macrophage polarization.

The mechanisms of pathogen-mediated TLR activation, signalling, and downstream inflammatory responses have been extensively investigated. The 10 TLRs expressed in humans either as monomers or through assembly of heterodimers signal through MyD88-dependent and MyD88-independent pathways that converge on activation of transcription factors. In particular, LPS-mediated activation of TLR4 in association with MD2 stimulates the phosphorylation (and activation) of the MAPKs p38 and c-Jun N-terminal kinase and activation of NF-*κ*B-dependent gene transcription [38]. Accordingly, by suppressing specifically the signalling mediated by the intracellular but not the extracellular domain of TLR4, CLI-095 restored both LPS-induced I*κ*B-*α* degradation and p38 phosphorylation [39]. We observed that pretreatment with GG9 interfered specifically with the p38-MAPK pathway, whereas GG6 significantly prevented I*κ*B-*α* degradation required for NF-*κ*B activation. This is consistent with a differential structure-activity relationship for the diaryl-hepta-1,4,6-trien-3-one scaffold of GG6 and the pyrazole analogue GG9 described in primary rat microglia [13], where GG9 does not modulate LPS-mediated TLR4-MD2 signalling. Here, we demonstrated different pharmacological profiles for the two analogues in disrupting or redirecting LPS signalling in human macrophages. In contrast to rat microglia [13] and other cell types [10, 40–42] where higher concentrations may have been used, curcumin affected neither LPS-induced signalling nor IL-1*β* release in human monocyte-derived macrophages. This could be due to species differences in TLR expression pattern as well as TLR4 and MD2 amino acid similarity and structural features [43–45], thus resulting in reduced curcumin potency in human versus rodent cells. However, the lack of activity of curcumin compared to its bisdemethoxy-derivative GG6 remains unexplained. It should be considered that GG6, due the absence of the methoxy functions peculiar of curcumin, has a more hydrophilic character compared to curcumin, as the hydroxyl functions on the side aryl rings are not involved in intramolecular hydrogen bonding with the adjacent methoxyls. Hence, this behaviour may account for a superior chemical stability of GG6 [46].

Functional assay of inhibition of LPS-induced cytokine production revealed additional differences in the pharmacological profile of GG6 and GG9. Abe et al. [47] first demonstrated that curcumin inhibits inflammatory cytokine production in human peripheral blood monocytes. However, we were unable to show any such effects of curcumin in macrophages, and the negative trend of GG6 on limiting LPS-induced IL-1*β* release did not achieve statistical significance. Human macrophages in the absence of inflammasome activators secrete considerably less IL-1*β* than primary rat microglia upon stimulation with LPS [9], thereby making it more difficult to assess the potential effects of pharmacological inhibitors. Furthermore, we were unable to identify any potential correlation of IL-1*β* data for GG6 (and curcumin) with their effects on the M1 immunophenotype because, as noted above, these compounds were unsuitable for flow cytometric assay. However, although polarized activation protocols are critical in shaping immunophenotype, functional assays are required when investigating pharmacological targeting of macrophages. GG9 was as effective as the TLR4 signalling inhibitor CLI-095 in reducing LPS-triggered IL-1*β* production, even if GG9 did not affect the NF-*κ*B pathway activated by TLR4 (LPS; Figure 4) or TLR3 (Poly(I:C) [48]) ligands. Given that IL-1*β* occurs in cells as both a largely nonsecreted proform and a cleaved and releasable mature form [49] and that the p38 MAPK pathway is involved in IL-1*β* secretion in human macrophages [50], this mechanism (Figure 5) may account for the observed downregulation of LPS-stimulated synthesis and release of IL-1*β* by GG9. Because the production of IL-1*β* is regulated by a two-step mechanism [51], GG9 may also have prevented IL-1*β* maturation, as described for curcumin in THP-1 cells [12]. To support the potential involvement of the NLRP3 inflammasome in GG9 action, we showed that this agent had no effect on the cell accumulation of TNF-*α*, which is independent from inflammasome activation [52].

Overall, these results suggest differential structural interactions of GG6 and GG9 at the receptor level. Our interpretation is that the diaryl-hepta-1,4,6-trien-3-one scaffold of GG6 interfered with LPS-induced signal transduction mainly via the TLR4/MD2 complex and downstream activation of NF-*κ*B (Figure 4; see also [13]), whereas GG9 impacted on partially overlapping, yet distinct, pathways possibly including MyD88-independent TLR4 [53] and/or TLR2 signalling (Figure 7). In fact, GG9 interfered with TLR2/TLR6 signalling that is coupled to p38 MAPK activation [54] and possibly with the calcium-dependent calmodulin-dependent kinase-Pyk2-extracellular signal-regulated kinase signalling pathway activated by zymosan in human blood-derived macrophages [55]. TLR2 expression is elevated in macrophages within inflamed human tissue [56], and our data support the relevance of TLR2 in inflammatory responses *in vivo*. Finally, an intriguing possibility, albeit supported by limited evidence, is that TLR4 associates with TLR2 to form TLR2-TLR4 heterodimers [57], which might finely tune LPS signalling and possibly expand the ligand spectrum.

## 5. Conclusion

Collectively, these results support the hypothesis that curcumin derivatives affect human monocyte-macrophage immunophenotypes and function by disrupting LPS signalling. The two analogues GG6 and GG9, structurally characterized by the same side aryl rings and a different core, showed a differential pharmacological profile through inhibition of the NF-*κ*B or p38 MAPK pathways, respectively, possibly resulting from binding to different TLR isoforms. Promoting a macrophage-protective phenotype has become a therapeutic goal in the treatment of inflammatory conditions, and the identification of factors that control cell activation is currently an area of active research. The present findings suggest that the curcumin analogues GG6 and GG9 represent promising lead compounds targeting immune cell activation.

## Abbreviations

APC: Allophycocyanin
CSF: Colony-stimulating factor
FBS: Fetal bovine serum
FITC: Fluorescein isothiocyanate
IL: Interleukin
IFN: Interferon
LPS: Lipopolysaccharide
MAPK: Mitogen-activated protein kinase
NF: Nuclear factor
PE: Phycoerythrin
RPMI: Roswell Park Memorial Institute medium
TLR: Toll-like receptor.

## Supplementary Materials

Supplementary Materials Supplemental Figure 1: human blood-derived monocytes were grown in RPMI + 10% FBS and differentiated into macrophages in the presence of 20 nM CSF-1 for 7 days at 37°C and 5% CO_2_. At the end of differentiation, macrophage viability was measured by MTT assay following treatment with curcumin, GG6, and GG9 (2.5–10 *μ*M) for 24 h. Data are expressed as mean raw absorbance values (±SEM) of 3 independent experiments performed in triplicate. Supplemental Figure 2: effects of curcumin, GG6, GG9, and CLI-095 on IL-1*β* accumulation in LPS-stimulated human macrophages. After differentiation, macrophages were pretreated with vehicle, curcumin, GG6, GG9, and CLI-095 as indicated for 30 min followed by stimulation with 1 *μ*g/ml LPS for 24 h in FBS-free RPMI in the presence of brefeldin (10 *μ*M) over the last 4 h to disrupt cytokine secretion pathways. Cell lysates were collected and analyzed for IL-1*β* release by ELISA. Bar graph represents the mean (±SEM) of 6 independent experiments. ^#^ *p* < 0.05 versus LPS. Supplemental Figure 3: human NLRP3 mRNA levels in macrophages. cDNA from resting, LPS-stimulated M1, and test agent-pretreated M1 macrophages were assayed by qPCR after 90-min LPS incubation. GAPDH served as loading control. Bars represent the mean (±SEM) of 3 independent experiments. Supplemental Figure 4: effects of GG9 and CLI-095 on cell-bound TNF-*α* expression in LPS-stimulated macrophages. After differentiation, macrophages were pretreated with either GG9 or CLI-095 for 30 min, and then incubated with LPS for 6 h at 37°C in RPMI + 10% FBS in the presence of brefeldin (10 *μ*M) over the last 4 h to disrupt cytokine secretion pathways. Prior to flow cytometry analysis, fixed/permeabilized cells were stained with anti-TNF-*α* antibodies (eBioscience). Fluorescence plots are from representative experiments (panel A), and bar graphs represent the mean (±SEM) of 5 independent experiments (panel B). ^∗^ *p* < 0.05 versus resting; ^#^ *p* < 0.05 versus LPS. In preliminary experiments, treatment with curcumin, GG6, or GG9 at concentrations tested in previous work (2.5–10 *μ*M) [11, 13] for 24 h did not compromise viability of human monocyte-derived macrophages as measured by the MTT assay (Figure S1). LPS-induced cellular accumulation of IL-1*β* was significantly reduced by pretreatment with both 7.5 *μ*M GG9 and 1 *μ*M CLI-095 as measured by ELISA (Figure S2). This was associated with a trend towards increased NLRP3 mRNA levels (Figure S3). In addition, GG9 had no effect on TNF-*α* cellular accumulation, which is independent from inflammasome activation (Figure S4).
