# Supplementary material for: Bisdemethoxycurcumin and Its Cyclized Pyrazole Analogue Differentially Disrupt Lipopolysaccharide Signalling in Human Monocyte-Derived Macrophages
Source: Mediators Inflamm. 2018 Feb 8;2018:2868702. doi: 10.1155/2018/2868702 (PMC5822910; doi:10.1155/2018/2868702)

Bis-demethoxycurcumin and its cyclized pyrazole analogue differentially disrupt lipopolysaccharide signaling in human monocyte-derived macrophages

Supplemental material - Methods

Cell viability assay

At the end of differentiation, macrophages seeded in 48-well plates were washed and treated with increasing concentrations (2.5-10 μM) of curcumin or the analogues GG6 or GG9 in RPMI 1640 + 10% FBS (Zusso et al., 2017). After 24 h incubation, 10 μL (3-(4,5-dimethylthiazol-2-yl)-2,5-diphenyltetrazolium bromide (MTT) stock solution (5 mg/mL) was added to each well for 4 h. At the end of incubation the formazan crystals were dissolved in 100 μL dimethyl sulfoxide (DMSO). MTT reduction was quantifed on a microplate reader (Victor2 Multilabel Counter, Wallac, Cambridge, MA, USA) using a test wavelength of 570 nm and a reference wavelength of 630 nm. Results were expressed as raw optical density (O.D.) values.

Gene expression analysis

Macrophages were lysed immediately after the 24-hour polarization step using RNeasy Plus Mini Kit (Qiagen, Switzerland). cDNA was generated using RevertAid Reverse Transcriptase and random primers according to the manufacturer’s instructions. qRT-PCR reactions were then run with Max SYBR Green PCR Master Mix on a CFX96 Real-Time PCR Detection System

thermocycler (Biorad, Milan, Italy). Primer pairs for human NLRP3 were designed ex novo using NCBI Primer-BLAST: Forward-AAGAGCCTTGCCCAGACATC; Reverse- AGCTTCAAACGACTCCCTGG. Results were normalized using the housekeeping gene GAPDH (Forward-CACCATCTTCCAGGAGCGAG; Reverse- CCTTCTCCATGGTGGTGAAGAC) and the ΔΔ cycle threshold method, and are expressed as relative fold of stimulated over control group, used as calibrator.

Legend to Supplemental Figures

Supplemental Figure 1: Human blood-derived monocytes were grown in RPMI + 10% FBS and differentiated into macrophages in the presence of 20 nM CSF-1 for 7 days at 37°C and 5% CO_2. At the end of differentiation, macrophage viability was measured by MTT assay following treatment with curcumin, GG6 and GG9 (2.5-10 µM) for 24 h. Data are expressed as mean raw absorbance values (±SEM) of 3 independent experiments performed in triplicate.

Supplemental Figure 2: Effects of curcumin, GG6, GG9 and CLI-095 on IL-1β accumulation in LPS-stimulated human macrophages. After differentiation, macrophages were pre-treated with curcumin, GG6, GG9 and CLI-095 as indicated for 30 min followed by stimulation with 1 µg/ml LPS for 24 h in FBS-free RPMI in the presence of brefeldin (10 µM) over the last 4 h to disrupt cytokine secretion pathways. Cell lysates were collected and analyzed for IL-1β release by ELISA. Bar graph represents the mean (±SEM) of 6 independent experiments. *p<0.01 *vs* resting, °p<0.05 *vs* LPS.

Supplemental Figure 3: Human NLRP3 mRNA levels in macrophages. cDNA from resting, LPS-stimulated M1 and test agent-pretreated M1 macrophages were assayed by qPCR after 90-min LPS incubation. GADPH served as loading control. Bars represent the mean (± SEM) of 3 independent experiments.

Supplemental Figure 4: Effects of GG9 and CLI-095 on cell-bound TNF-α expression in LPS-stimulated macrophages. After differentiation, macrophages were pre-treated with either GG9 or CLI-095 for 30 min, and then incubated with LPS for 6 h at 37°C in RPMI + 10% FBS in the presence of brefeldin (10 µM) over the last 4 h to disrupt cytokine secretion pathways. Prior to flow cytometry analysis fixed/permeabilized cells were stained with anti-TNF-α antibodies (eBioscience). Fluorescence plots are from representative experiments (panel **A**), and bar graphs represent the mean (± SEM) of 5 independent experiments (panel **B**). * $p < 0.05$ vs. resting; # $p < 0.05$ vs. LPS.

.

Supplemental Fig. 1

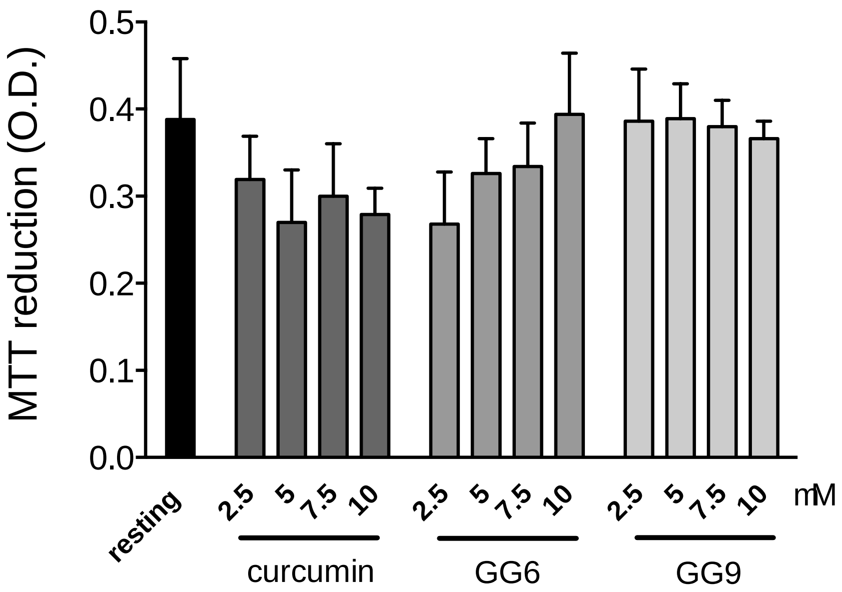

Supplemental Fig. 2

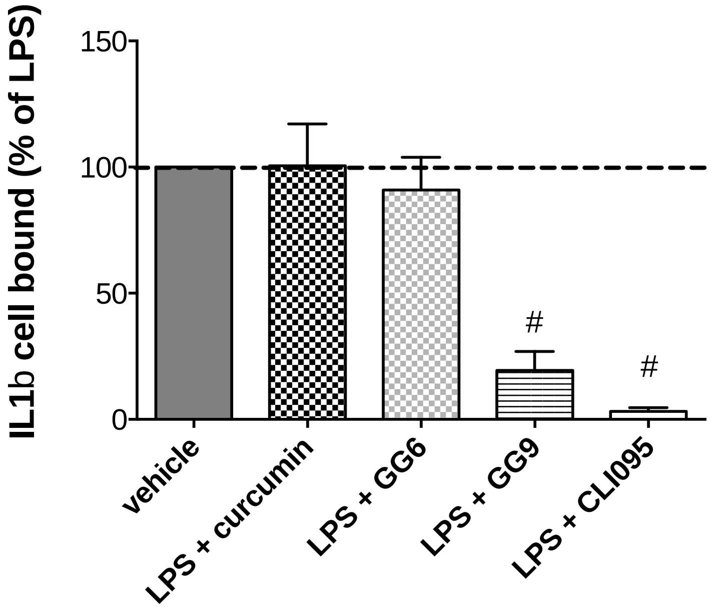

Supplemental Fig. 3

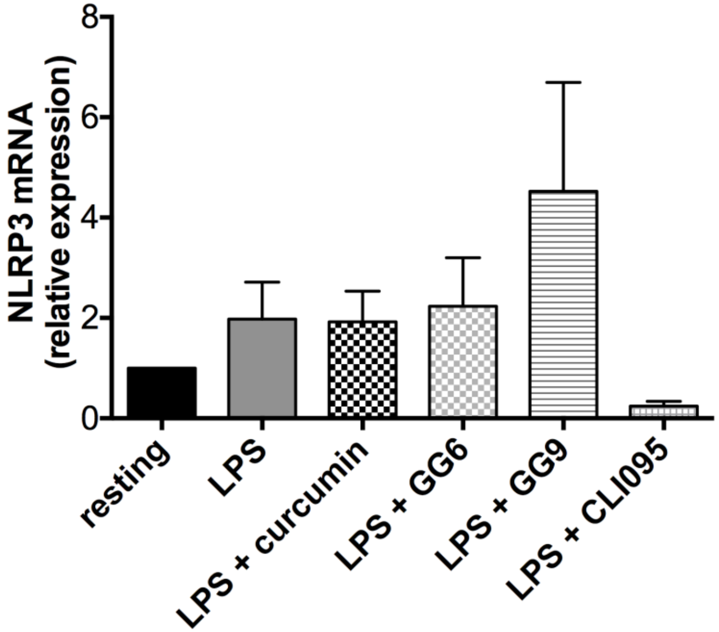

Supplemental Fig. 4

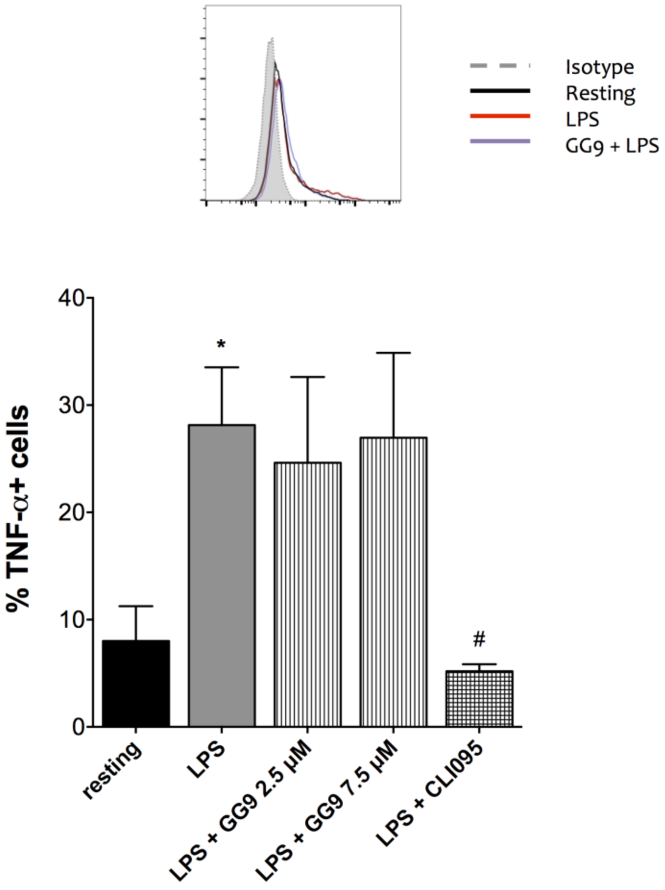

Supplement: Supplementary Materials — Supplemental Figure 1: human blood-derived monocytes were grown in RPMI + 10% FBS and differentiated into macrophages in the presence of 20 nM CSF-1 for 7 days at 37°C and 5% CO2. At the end of differentiation, macrophage viability was measured by MTT assay following treatment with curcumin, GG6, and GG9 (2.5–10 μM) for 24 h. Data are expressed as mean raw absorbance values (±SEM) of 3 independent experiments performed in triplicate. Supplemental Figure 2: effects of curcumin, GG6, GG9, and CLI-095 on IL-1β accumulation in LPS-stimulated human macrophages. After differentiation, macrophages were pretreated with vehicle, curcumin, GG6, GG9, and CLI-095 as indicated for 30 min followed by stimulation with 1 μg/ml LPS for 24 h in FBS-free RPMI in the presence of brefeldin (10 μM) over the last 4 h to disrupt cytokine secretion pathways. Cell lysates were collected and analyzed for IL-1β release by ELISA. Bar graph represents the mean (±SEM) of 6 independent experiments. # p < 0.05 versus LPS. Supplemental Figure 3: human NLRP3 mRNA levels in macrophages. cDNA from resting, LPS-stimulated M1, and test agent-pretreated M1 macrophages were assayed by qPCR after 90-min LPS incubation. GAPDH served as loading control. Bars represent the mean (±SEM) of 3 independent experiments. Supplemental Figure 4: effects of GG9 and CLI-095 on cell-bound TNF-α expression in LPS-stimulated macrophages. After differentiation, macrophages were pretreated with either GG9 or CLI-095 for 30 min, and then incubated with LPS for 6 h at 37°C in RPMI + 10% FBS in the presence of brefeldin (10 μM) over the last 4 h to disrupt cytokine secretion pathways. Prior to flow cytometry analysis, fixed/permeabilized cells were stained with anti-TNF-α antibodies (eBioscience). Fluorescence plots are from representative experiments (panel A), and bar graphs represent the mean (±SEM) of 5 independent experiments (panel B). ∗ p < 0.05 versus resting; # p < 0.05 versus LPS. In preliminary experiments, treatment [file 2868702.f1.pdf]
